# Prior knowledge-based precise diagnosis of blend sign from head computed tomography

**DOI:** 10.3389/fnins.2023.1112355

**Published:** 2023-02-10

**Authors:** Chen Wang, Jiefu Yu, Jiang Zhong, Shuai Han, Yafei Qi, Bin Fang, Xue Li

**Affiliations:** ^1^College of Computer Science, Chongqing University, Chongqing, China; ^2^Department of Neurosurgery, The First Hospital of China Medical University, Shenyang, China; ^3^Department of Neurosurgery, Shengjing Hospital of China Medical University, Shenyang, China; ^4^College of Computer Science and Engineering, Central South University, Changsha, China; ^5^School of Information Technology and Electrical Engineering, The University of Queensland, Brisbane, QLD, Australia

**Keywords:** blend sign, intracranial hemorrhage, hemorrhage expansion, prior knowledge, self-knowledge distillation, convolutional neural network

## Abstract

**Introduction:**

Automated diagnosis of intracranial hemorrhage on head computed tomography (CT) plays a decisive role in clinical management. This paper presents a prior knowledge-based precise diagnosis of blend sign network from head CT scans.

**Method:**

We employ the object detection task as an auxiliary task in addition to the classification task, which could incorporate the hemorrhage location as prior knowledge into the detection framework. The auxiliary task could help the model pay more attention to the regions with hemorrhage, which is beneficial for distinguishing the blend sign. Furthermore, we propose a self-knowledge distillation strategy to deal with inaccuracy annotations.

**Results:**

In the experiment, we retrospectively collected 1749 anonymous non-contrast head CT scans from the First Affiliated Hospital of China Medical University. The dataset contains three categories: no intracranial hemorrhage (non-ICH), normal intracranial hemorrhage (normal ICH), and blend sign. The experimental results demonstrate that our method performs better than other methods.

**Discussion:**

Our method has the potential to assist less-experienced head CT interpreters, reduce radiologists' workload, and improve efficiency in natural clinical settings.

## Introduction

**Figure 1:**
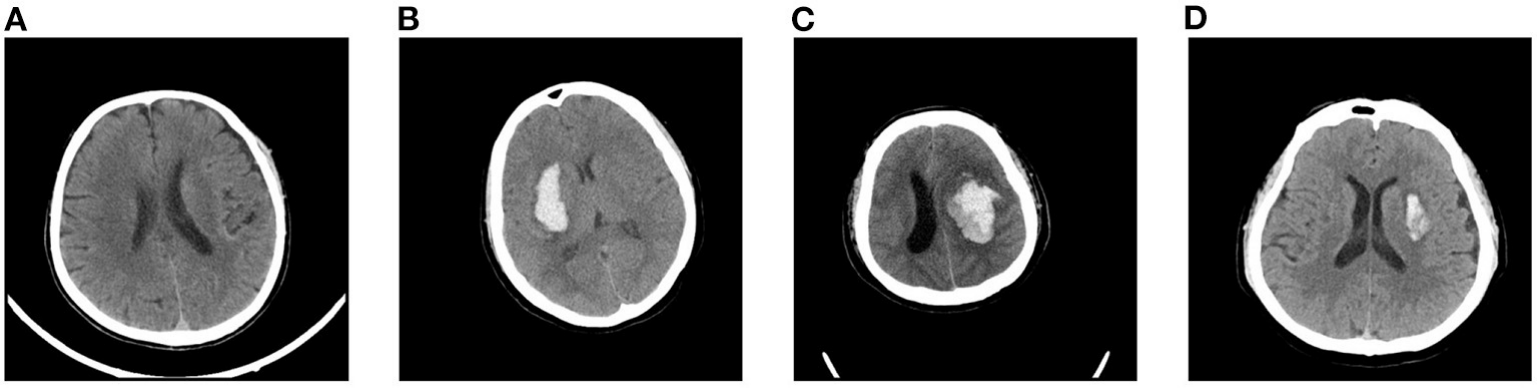
Examples of slices in our dataset: **(A)** non-ICH, **(B)** normal ICH, **(C)** blend sign, and **(D)** blend sign mimic (Normal ICH).

Intracranial hemorrhage (ICH) is a serious neurological disorder. It accounts for about 30% of the whole number of patients with stroke (Qureshi et al., 2009). Many factors such as congenital development, vascular disease, and head injury could lead to ICH (Heit et al., 2017). According to the hemorrhage location, some recent studies (Qureshi et al., 2001) subdivide ICH into intraparenchymal hemorrhage (IPH), intraventricular hemorrhage (IVH), epidural hemorrhage (EDH), subdural hemorrhage (SDH), and subarachnoid hemorrhage (SAH) (Qureshi et al., 2001). Recently, some researchers have paid much attention to the blend sign and black hole sign, two new types of ICH (Li et al., 2015, 2016). Blend sign (Li et al., 2015) is composed of two parts with apparently different CT attenuation. There is a well-defined margin (Li et al., 2017) between the hyperattenuated and relatively hypoattenuated regions, as shown in Figure 1. Some recent studies (Yagi et al., 2019; Zhang et al., 2020; Li et al., 2021; Yang et al., 2021) have shown that blend sign and black hole sign are closely associated with hemorrhage expansion in ICH.

Non-contrast head computed tomography (CT) is a well-known and practical imaging approach for the diagnosis of intracranial hemorrhage (Heit et al., 2017). In the non-contrast CT slices, regions with intracranial hemorrhage appear highlighted since blood has a slightly higher density than other brain tissues (Nguyen et al., 2016). Patients in emergency departments usually need an evaluation of head CT. In general, the precise diagnosis of intracranial hemorrhage is crucial in patients to assess the need for clinical treatment (Chilamkurthy et al., 2018). Most hospitals usually provide CT scan interpretations by junior radiologists or emergency physicians. Then some senior radiologists will review the initial interpretations. The CT scan interpretation is time-consuming, of low quality, and is unreliable. Several studies have confirmed that some misinterpretations may even lead to clinical consequences (Alfaro et al., 1995; Strub et al., 2007). A precise diagnosis system for intracranial hemorrhage from head CT scans is desirable.

Artificial intelligence and deep learning have recently shown great performance in medically assisted diagnosis (Shin et al., 2016; Havaei et al., 2017; Kamnitsas et al., 2017; Chen et al., 2018). Chen et al. (2018) presented a 3D U-Net to segment cranial vasculature in CTA volume without manual annotations. Havaei et al. (2017) proposed a brain tumor segmentation model with deep neural networks. Kamnitsas et al. (2017) provided an efficient multi-scale 3D CNN with fully connected CRF for accurate brain lesion segmentation. Shin et al. (2016) utilized convolutional neural networks for computer-aided detection problems. Recently, some researchers have introduced generative learning into brain disease diagnosis (Wang et al., 2018, 2022; Hu et al., 2019, 2021; Yu et al., 2021, 2022; You et al., 2022). Wang et al. (2018) presented a convolutional neural network-based framework for bone age assessment. Hu et al. (2019) proposed one adversarial U-Net with different normalizations for cross-modality synthesis from MRI to PET. Yu et al. (2021) applied GAN with high-order pooling for Alzheimer's disease. Yu et al. (2022) introduced a novel multi-directional perception generative adversarial network to visualize the morphological features of Alzheimer's disease. Hu et al. (2021) introduced bidirectional mapping generative adversarial networks for brain MRI to PET synthesis. You et al. (2022) designed a fine perceptive generative adversarial network to produce high-resolution MR images from low-resolution counterparts in the wavelet domain. Wang et al. (2022) proposed a segmentation model for brain stroke lesions with consistent perception generative adversarial networks.

Some researchers have explored the detection of abnormalities in head CT with machine learning and deep learning methods (Xiao et al., 2010; Li et al., 2012; Chang et al., 2018; Titano et al., 2018). Classification-based approach is the conventional approach. Li et al. (2012) reported a machine-learning algorithm with high diagnostic value for SAH detection. Prevedello et al. (2017) proposed one small deep-learning model to detect critical test findings for head CT. Chilamkurthy et al. (2018) proposed a deep-learning algorithm for detecting critical findings in head CT scans. They retrospectively collected 4,304 scans for evaluation. Ye et al. (2019) introduced a three-dimensional (3D) joint convolutional neural network for the classification of five subcategories of ICH. Lee et al. (2019) presented an explainable deep-learning algorithm for ICH classification with a small dataset. One disadvantage of these classification-based methods is that the model may fit some unimportant features, such as the background. Moreover, some researchers (Grewal et al., 2018; Liu et al., 2021) introduced the segmentation into the diagnosis of head CT. Grewal et al. (2018) applied three segmentation tasks as auxiliary tasks to guide the classification model's attention to the regions with hemorrhage. Kuo et al. (2019) proposed an expert-level detection model for acute intracranial hemorrhage, which performed joint classification tasks and segmentation tasks. Liu et al. (2021) presented a few short-learning model for intracranial hemorrhage segmentation model with classification task as the auxiliary task. These studies show that segmentation tasks can help highlight the regions with hemorrhage and extract more discriminative features (Xie et al., 2020). However, the segmentation task relies on highly accurate pixel-level annotations, which are time-consuming and challenging for large scale datasets. Furthermore, some researchers directly applied the object detection models for the diagnosis of hemorrhage. Chang et al. (2018) collected 11,021 CT scans from a single institution and proposed a hybrid convolutional neural network (CNN) with mask RCNN (He et al., 2017) for ICH detection and quantification. However, the regions with hemorrhage are fewer in the CT scans, and most CT scans have no hemorrhage. These anchor-based detection methods face the imbalance problem of samples and are always difficult for model optimization.

In fact, the region with hemorrhage is the most important basis for distinguishing blend sign from normal ICH. Radiologists mainly make evaluations based on the region with hemorrhage (Li et al., 2017). Then we employ the region with hemorrhage as a prior knowledge, and hope to add this prior knowledge to the model. This study proposes a prior knowledge-based model for the precise diagnosis of blend sign from head CT scans. We apply object detection to replace segmentation as the auxiliary task to reduce the annotation difficulty. Object detection only needs region-level annotation, e.g., center and bounding box, which are simpler and more robust than segmentation. Furthermore, there are inevitably some inaccuracies in the annotations. We propose a self-knowledge distillation strategy to deal with the inaccuracy annotations. We train a model as the teacher model and generate the pseudo labels for the training images. The pseudo labels contain much information about the negative categories. Using the pseudo labels as the supervision can gradually reduce the impact of inaccuracy annotations. Finally, we evaluated the proposed model on the collected dataset. Extensive results show that our method achieves better performance than the baseline model.

The contributions of this study are 3-fold:

- We retrospectively collected 1,749 anonymous non-contrast head CT scans from the First Affiliated Hospital of China Medical University and annotated 13,276 slices for evaluation. These slices are divided into three categories: no intracranial hemorrhage (non-ICH), normal intracranial hemorrhage (normal ICH), and blend sign.
- We present a prior knowledge-based diagnosis of blend sign from head CT scans. We apply an object detection task as the auxiliary task to reduce the annotation difficulty. The auxiliary task can help the model pay more attention to the regions with hemorrhage.
- We propose a self-knowledge distillation strategy to deal with incorrect annotations. The soft predictions contain much information about the negative categories and can gradually reduce the impact of inaccuracy annotations.

## Materials and methods

### Data collection and labeling

**Figure 2:**
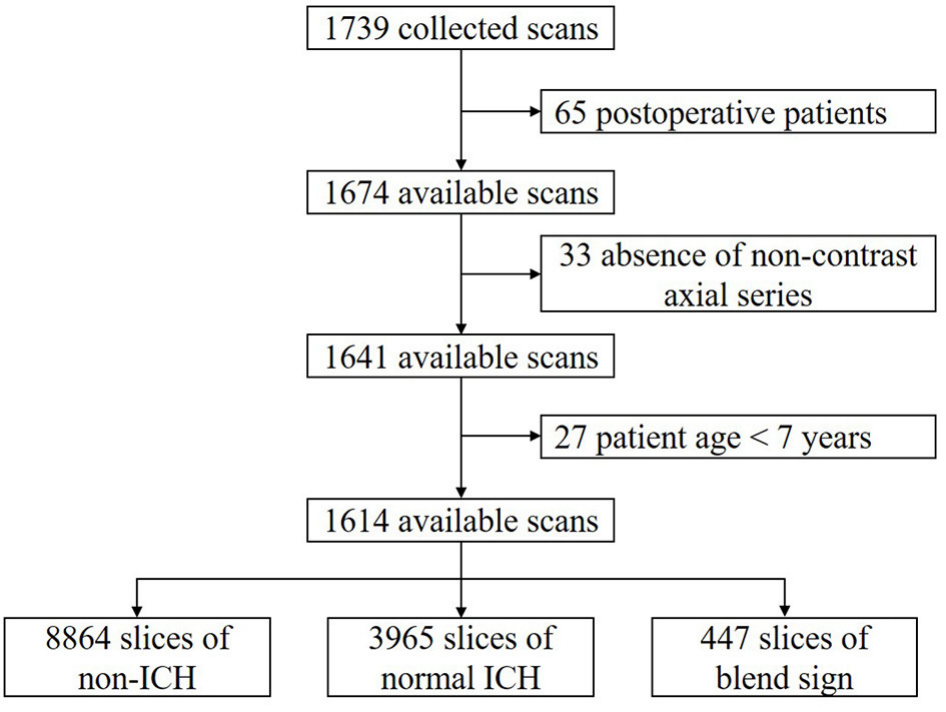
Dataset collection and selection process.

We retrospectively collected 1,749 anonymous non-contrast head CT scans from the Department of Neurosurgery, the First Hospital of China Medical University, Shenyang, China. The time of data collection is from 19 September 2018 to 24 December 2020. All scans were from the Asian population. CT scanners used in our dataset had slices per rotation varying from 16 to 128. All of the CT scans in our dataset were independently annotated at scan level and slice level by three radiologists. These radiologists had corresponding experiences of 6, 11, and 15 years in interpreting head CT scans. None of them was involved in the clinical care of the enrolled patients. After careful review and annotation, 125 CT scans were then excluded from further analysis due to the following reasons: postoperative patients (65); absence of non-contrast axial series (33); and patients were younger than 7 years (27). The remaining 1,614 available CT scans were finally used in our study. The dataset collection and selection process is shown in Figure 2.

Each of the experienced radiologists independently evaluated the scans and slices in our dataset. For the classification task, experienced radiologists recorded the following findings for each slice (Chilamkurthy et al., 2018): (1) normal head CT (non-ICH), (2) normal ICH (including EDH, SDH, SAH, IPH, and IVH), (3) blend sign. In some slices, both normal ICH and blend sign may occur in the same slices, and we consider these slices as blend sign slices. For the detection task, each radiologist records whether one slice contains hemorrhage or not. For each hemorrhage region, the experienced radiologists annotate the center point and its bounding box (left, right, top, and bottom).

We apply the majority vote of these three radiologists' interpretations as the gold standard (Chilamkurthy et al., 2018). By slice-level annotation, there were 8,864 slices with non-ICH, 3,965 slices with normal ICH, and 447 slices with blend sign. The ratio of blend sign: normal ICH:non-ICH is approximately equal to 1:9:22. To effectively evaluate our algorithm as well as benefit the learning process, we intentionally kept such a high prevalence of blend sign in our dataset to ensure that there were enough positive samples.

#### Data pre-processing and augmentation

To highlight and emphasize specific pixels, we choose three different windows and encode them into the following RGB images: tissue window (WL = 40, WW = 40) for the red channel; brain window (WL = 50, WW = 100) for the green channel; and blood window (WL = 60, WW = 40) for the blue channel. Before being fed into the model, we reshape all CT slices to size 512×512 to reduce GPU memory usage. Then we convert all pixels from CT slices into floating point tensors and rescale the pixels values (between 0 and 255) into the [0, 1] interval.

**Figure 3:**
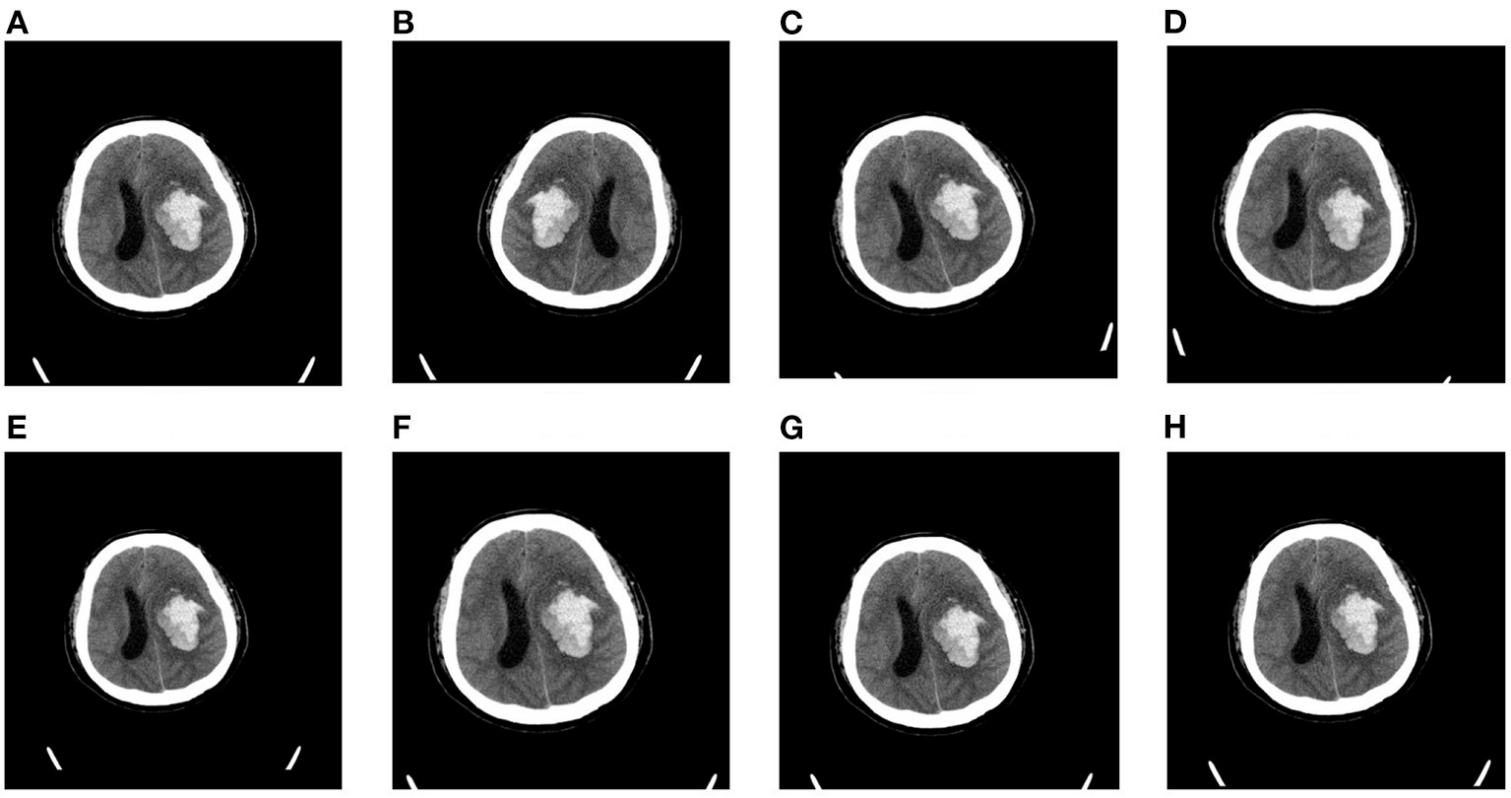
Random augmentations for CT slices: **(A)** original slice, **(B)** left-right flipping, **(C)** rotation (10°, **(D)** rotation (−10°, **(E)** scaling (0.9), **(F)** scaling (1.1), **(G)** shifting (right), and **(H)** shifting (down).

Considering that our dataset is relatively small, we apply data augmentation to mitigate over-fitting in our task. In this article, we choose five forms of data augmentation operations, left-right flipping, left-right shifting, up-down shifting, random rotations (up to 10 degrees), and random scaling (0.9 to 1.1). The augmentation operations are shown in Figure 3. During the training process, the data generator will randomly choose the above augmentation operations for each slice, which means that the input to the model is different at each epoch. We find that data augmentation could largely enrich the training dataset and improves the performance of our model on the task of blend sign and normal ICH detection.

#### Overview of the proposed method

**Figure 4:**
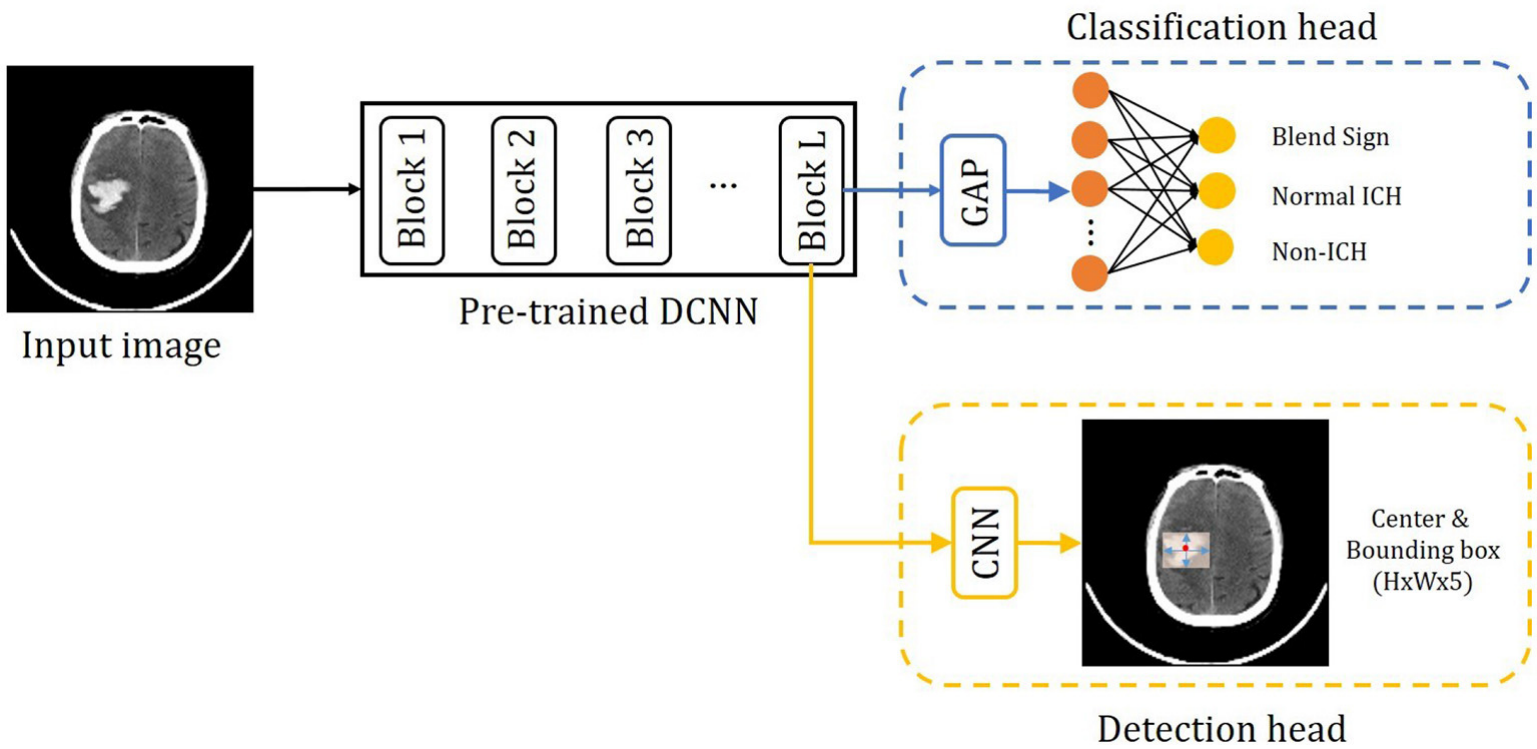
Overview of the precise diagnosis of the blend sign system. Given one CT slice, we first use a pre-trained CNN (trained on the ImageNet dataset) to extract feature maps, then put the feature maps into the classification branch and the segmentation branch, respectively.

The proposed method consists of three parts: the pre-trained DCNN, the classification branch, and the detection branch. The pipeline of the proposed method is shown in Figure 4. The input image is fed into the pre-trained DCNN to extract the feature maps. Then the feature maps from the last convolutional block (Block L) are fed into two branches, the classification branch and the detection branch. The classification branch gets the predictions with global average pooling (GAP) layer and linear layers. Then detection branch gets the locations with convolutional layers. In this way, the detection branch could transfer the hemorrhage localization information to boost the classification branch.

#### Pre-trained networks

Considering that our dataset is relatively small, we decided to use some pre-trained deep convolutional neural networks (DCNNs) as the backbones. Following (Lee et al., 2019), we choose one widely applied architecture, VGG16 (Simonyan and Zisserman, 2015), ResNet-50 (He et al., 2016), and Inception v3 (Szegedy et al., 2016), as the pre-trained networks. These architectures are pre-trained on one subset of ImageNet dataset (Deng et al., 2009). For the classification branch, we add one GAP layer and one linear layer after the last convolutional block (block L). We add two convolutional layers for the detection branch after the last convolutional block ( Block L). Then we fine-tune the pre-trained backbone, the classification branch, and the detection branch with our dataset.

#### Classification branch

Automatic blend sign classification is a multi-class classification problem. Each input image can be labeled as three mutually exclusive types. For each input slice, the classification branch will produce a three-dimensional output vector, where output_i_ is the probability that the input slice belongs to class i. The conventional loss is cross-entropy (CE) loss as follows:


(1) $$L_{ce}=-\sum_{\text{i}=1}^{3}y^{i}\ln{\hat{y}}^{i}$$

Where *y*^*i*^ is the ground truth and ${\hat{y}}^{i}$ is the probability of class i our model predicts given an input slice *x*.

Considering the class-imbalanced problem in our dataset, models trained on these samples are biased toward dominate classes, non-ICH and normal ICH. To deal with the class-imbalanced problem, we try to use weighted cross-entropy (WCE) loss function as:


(2) $$\begin{array}{l} L_{wce}=-\overset{3}{\underset{i=1}{\sum }}w_{i}y^{i}\ln{\hat{y}}^{i} \end{array}$$

Where *w*_*i*_ is the corresponding loss weight for class i. The weights are to reduce the effects of imbalanced data distribution. The weights can be fixed or automatically adjusted during the training process. Through conductive experiment, we set the weights as *w*_1,2,3_ = 1, 2, 20, respectively. In the experiments, we find that WCE loss could partially solve the class-imbalanced problem and improve the performance of our algorithm.

#### Detection branch

The detection branch is inspired by the famous anchor-free detection frameworks, such as FCOS (Tian et al., 2019) and CenterNet (Duan et al., 2019). The detection branch aims to predict the localization of the hemorrhage region for the input image. In this branch, we consider the normal ICH and blend sign as the foreground, and consider the non-ICH as the background. The output of the detection branch is one of the key-point maps with five channels. The first channel indicates the probability of the center point of the hemorrhage region. The other four channels are the bounding box (left, right, top, and bottom) of the hemorrhage region. The loss function for the detection branch is defined as:


(3) $$\begin{array}{l} L_{det}=\frac{1}{N}\overset{N}{\underset{j=1}{\sum }}L_{cls}\left(p_{j},{\hat{p}}_{j}\right)+\frac{1}{N}\overset{N}{\underset{j=1}{\sum }}p_{j}L_{reg}\left(b_{j},b_{j}^{*}\right), \end{array}$$

(4) $$\begin{array}{l} L_{cls}\left(p_{j},{\hat{p}}_{j}\right)=p_{j}\ln{\hat{p}}_{j}+\left(1-p_{j}\right)\ln{\hat{p}}_{j}, \end{array}$$

(5) $$L_{reg}(b_{j},{\hat{b}}_{j})=\left|b_{j}-{\hat{b}}_{j}\right|,$$

Where *p*_*j*_ and ${\hat{p}}_{j}$ are the ground truth and probability of the *j*-th position being the center point of one hemorrhage region. The ground truth *p*_*j*_ is equal to 1 if the *j*-th position is the center point of one hemorrhage region, and *p*_*j*_ is equal to 0 if the *j*-th position is not the center point of one hemorrhage region. *b*_*j*_ represents the ground truth bounding box (*b*_*j,left*_, *b*_*j,right*_, *b*_*j,top*_, and *b*_*j,bottom*_) for the hemorrhage region, ${\hat{b}}_{j}$ is the predicted bounding box. *N* is the number of pixels in an input image. *L*_*cls*_ is log loss over two classes (center point vs. not center point). *L*_*reg*_ is the smooth *L*_1_ loss following (Tian et al., 2019). Following Duan et al. (2019), we perform Gaussian rendering for all ground truth *p* for faster training.

#### Self-knowledge distillation

**Figure 5:**
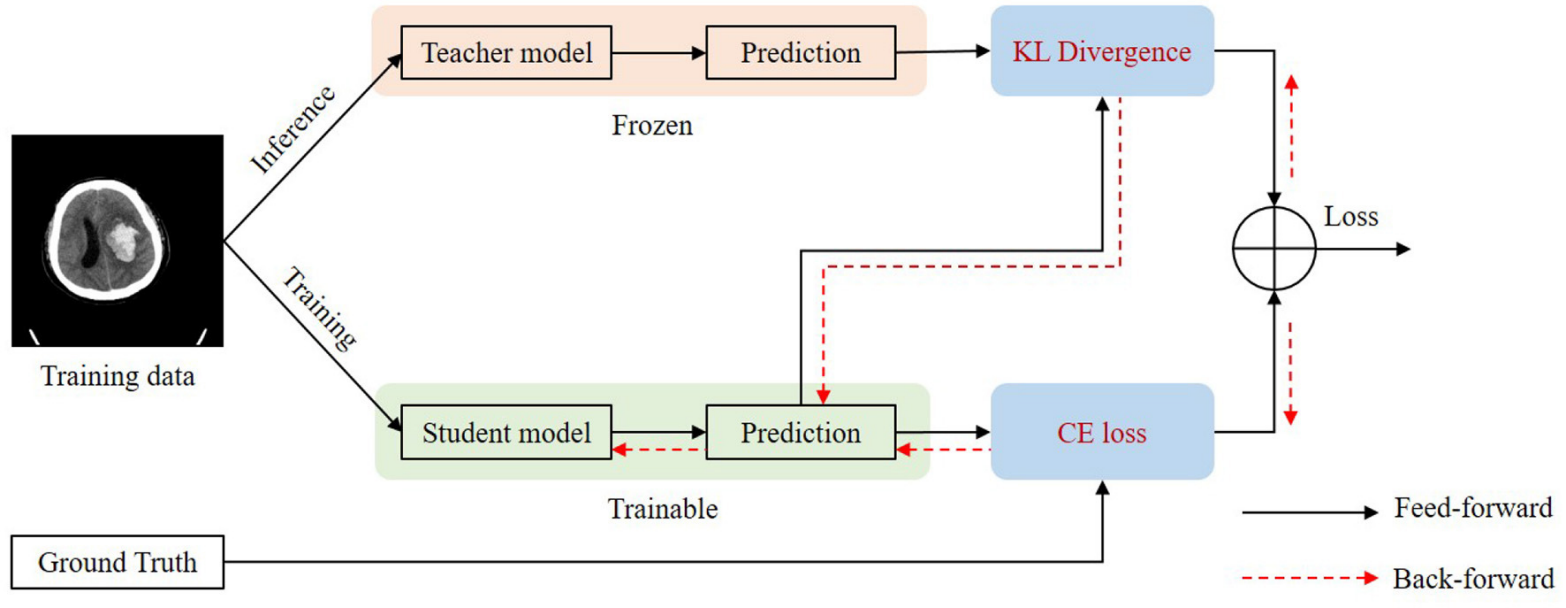
Illustration of self-knowledge distillation.

For some slices, it is difficult to distinguish blend sign and normal ICH. Thus, it will inevitably lead to some inaccurate labels. The inaccurate annotations may bring a certain degree of challenges for the training process and the model would be misled by these inaccurate labels. We applied a self-knowledge distillation (Zhang et al., 2021) strategy to solve this problem. In addition to the positive category, the predictions also contain a lot of information about the negative category. The information on the negative category can be transferred to the student by self-knowledge distillation. Thus, the inaccuracy annotations can be changed gradually. Figure 5 shows the training process of self-knowledge distillation. We first train a model with the training images as the teacher model. Then we use the teacher model to generate predictions (pseudo labels) for each training image. Next, we train a student model by minimizing the distance between the predictions from the teacher and the student as follows:


(6) $$L_{skd}=\text{KL}({\hat{y}}_{t}\|{\hat{y}}_{s}),$$

Where KL is the Kullback–Leibler divergence to measure the distance between two predictions. ${\hat{y}}_{t}$ and ${\hat{y}}_{s}$ are the predictions of the classification branch from the teacher and the student, respectively. Since inaccurate labels mainly affect the classification branch, we apply self-knowledge distillation for the classification task. We apply the student model as the teacher model in the next iteration of the training process.

#### Optimization

The whole loss function for the proposed method is as follows:


(7) $$\begin{array}{l} L=L_{wce}+{\text{λ}}_{1}L_{det}+{\text{λ}}_{2}L_{skd}, \end{array}$$

Where λ_1_ and λ_2_ are the hyper-parameters to balance the effect of different losses. Through conductive experiment, we set λ_1_ = 1 and λ_2_ = 10 for our experiments.

## Experiments and results

### Implementation details

#### Training details

We carry out all experiments on the PyTorch framework (version 1.7.1) with a single Nvidia GTX 1080Ti GPU card of 11 GB memory. We choose stochastic gradient descent (SGD) with the momentum (0.9) and the weight decay (0.0005) as the optimizer. We apply the “ploy” learning rate decay, in which the learning rate equals to $base_{-}lr*{\left(1-\frac{iter}{total_{-}iter}\right)}^{power}$. We set the base learning rate (*base*_−_*lr*) as 0.01 and the *power* as 0.9. The mini-batch size is 16, the input size is 512 × 512, and the training epoch number is 50. Data augmentation operations include random flipping, re-scaling (from 2 to 0.5), and rotation.

#### Evaluation metric

Considering that our dataset is with a class-imbalanced problem, we would like to choose *Sensitivity*, *Specificity*, *F*1 score, and AUC (area under the receiver operating characteristic (ROC) curve) as the statistical evaluation metrics. We define TP as the number of true positives, FP as the number of false positives, TN as the number of true negatives, and FN as the number of false negatives. The definition of sensitivity, specificity, and F1 score are as follows:


(8) $$\begin{array}{l} Sensitivity=\frac{TP}{TP+FN}, \end{array}$$

(9) $$\begin{array}{l} Specificity=\frac{TN}{TN+FP}, \end{array}$$

(10) $$\begin{array}{l} F1=\frac{2\times TP}{2\times TP+FN+FP}. \end{array}$$

ROC curves were obtained by varying the threshold and plotting the true positive rate (i.e., sensitivity) and false positive rate (i.e., 1-specificity) at each threshold. We performed all statistical analyzes with the python package scikit-learn, and generated all statistical plots with Matplotlib.

#### Evaluation protocol

Considering that our dataset is relatively small, we may have insufficient samples for validation and test set if we use simple hold-out validation. The evaluation results may also be not reliable. To address this problem, we apply *K*-fold cross-validation as the evaluation protocol. It will split the dataset into *K* partitions with equal size. For each fold, we choose one partition as the validation set and train a model on the remaining *K*-1 partitions. The final score would then be the average of *K* validation scores. Particularly, in this study, we use a 5-fold validation as the evaluation protocol. We will randomly split the dataset into five partitions of equal size, and then train and evaluate five different models. Then the final evaluation score is the average of five different evaluation scores.

## Results

Table 1 presents the comprehensive comparisons with existing datasets on five aspects: number of scans, five-type annotation, blend sign, and pixel-wise annotation. We can find that most datasets focus on the five-type (ICH, IPH, IVH, EDH, and SDH) detection tasks. These datasets rarely provide the pixel-wise annotation except for the dataset, as explained by in Grewal et al. (2018). This is because the pixel-wise annotation is time-consuming, which is challenging for large scale datasets. Our dataset is the only one to consider the detection of blend sign.

**Table 1:** Comparison with other related datasets for the detection of intracranial hemorrhage.

**Dataset**	**# Scans**	**Five-type**	**Pixel-wise**	**Blend**
		**annotation**	**annotation**	**sign**
Chilamkurthy et al. (2018)	491	✓	×	×
Grewal et al. (2018)	252	×	✓	×
Lee et al. (2019)	904	✓	×	×
Ye et al. (2019)	2,836	✓	×	×
Ours	1,614	×	×	✓

^#^Scans: number of scans in the dataset. Five-type annotation: annotation for ICH, IPH, IVH, EDH, and SDH.

Table 2 presents the inter-rater interpretation agreement among the three radiologists. Concordance between the three radiologists on our dataset was highers for non-ICH (All Fleiss's κ = 0.91), representing excellent agreement with these findings. Blend sign has the lowest concordance with All Fleiss's κ = 0.79, indicating substantial agreement.

**Table 2:** Reliability of the gold standards for our dataset.

	**R1 and R2**		**R2 and R3**		**R1 and R3**		**All Fleiss's κ**
	*p*(%)	κ	*p*(%)	κ	*p*(%)	κ	
Non-ICH	97	0.91	98	0.92	98	0.91	0.91
Normal ICH	94	0.85	96	0.87	95	0.86	0.86
Blend sign	89	0.78	92	0.81	91	0.79	0.79

R, radiologist; *p*, percentage agreement rate; κ, Cohen's κ coefficient, a statistic to measure inter-rater agreement; All Fleiss's κ coefficient, a statistic to measure the agreements among multiple raters. Values of κ and All Fleiss's κ >0.80 indicate excellent agreement, and 0.60–0.80 indicate substantial agreement.

Table 3 summarizes the performance of the proposed method. Our method achieved AUCs of 0.972 for blend sign, 0.978 for normal ICH, and 0.999 for non-ICH. For *Sensitivity*, our method achieved 0.845 for blend sign, 0.898 for normal ICH, and 0.984 for non-ICH. For *Specificity*, our method achieved 0.941 for blend sign, 0.936 for normal ICH, and 0.986 for non-ICH. Based on these results, we have three significant findings. First, the performance of normal ICH was consistently higher than that of the blend sign. This may be because, compared with the blend sign, slices with normal ICH are much easier to be discriminated. Second, auxiliary task (detection task) and self-knowledge distillation method could boost the performance, especially for the sensitivity of blend sign (from 0.544 to 0.845). This demonstrated the effectiveness of auxiliary task (detection task) and self-knowledge distillation method. Third, the sensitivity of normal ICH was slightly decreased from 0.929 to 0.898. This is because the model would predict some slices with normal ICH into blend sign. Table 4 shows the performance with different pre-trained backbones. We can observe that there are slight differences among different pre-trained backbones.

**Table 3:** Performance of the automated detection algorithm on our dataset with ResNet-50 as the backbone.

	**WCE**	**AT**	**SKD**	**Sensitivity**	**Specificity**	**F1 score**	**AUC**
	✓			0.544	**0.986**	0.672	0.966
Blend Sign	✓	✓		0.716	0.977	0.768	0.972
	✓	✓	✓	**0.845**	0.941	**0.781**	**0.977**
	✓			**0.929**	0.842	0.913	0.964
Normal ICH	✓	✓		0.897	0.897	0.911	0.967
	✓	✓	✓	0.898	**0.936**	**0.928**	**0.978**
	✓			0.978	0.953	0.932	0.996
Non-ICH	✓	✓		0.982	0.945	0.929	0.994
	✓	✓	✓	**0.984**	**0.986**	**0.968**	**0.999**

WCE, weighted cross-entropy loss; AT, auxiliary task (detection task); SKD, self-knowledge distillation. The bold values indicate the best score.

**Table 4:** Performance of the automated detection algorithm with different pre-trained backbones.

	**Backbone**	**F1 score**	**AUC**
	VGG16	0.762	0.964
Blend sign	ResNet-50	0.781	0.977
	Inception V3	0.773	0.970
	VGG16	0.932	0.981
Normal ICH	ResNet-50	0.928	0.978
	Inception V3	0.915	0.972
	VGG16	0.949	0.995
Non-ICH	ResNet-50	0.968	0.999
	Inception V3	0.955	0.997

### Error analysis

**Figure 6:**
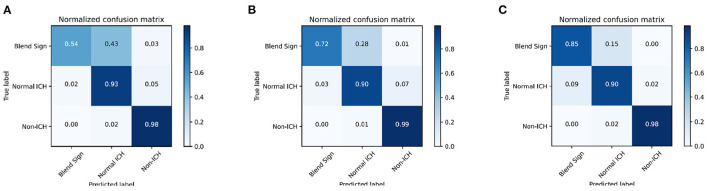
Confusion matrix of the classification results. **(A)** WCE, **(B)** WCE+AT, and **(C)** WCE+AT+SKD. WCE, weighted cross-entropy loss; AT, auxiliary task (detection task); SKD, self-knowledge distillation.

We introduce the confusion matrix to evaluate the performance and analyze the error. The row of the confusion matrix is the predicted label, and the column is the true label. Figures 6A–C shows the confusion matrix with different combinations. From Figure 6A, we find that the classification of blend sign is terrible, 43% of slices with blend signs were incorrectly predicted as non-ICH. Meanwhile, only 2% of slices with normal ICH were incorrectly predicted as blend sign. It is because the model is unable to distinguish the slices with normal ICH and blend sign. The model tends to predict the slice with a blend sign as normal ICH. In addition, we propose the auxiliary task (detection task) and find that only 28% of slices with blend sign were incorrectly predicted as non-ICH. The results indicate that the auxiliary task (detection task) can force the model to pay more attention to the regions with hemorrhage and make more accurate predictions. Moreover, when we apply the self-knowledge distillation, 85% of slices with blend sign were correctly predicted, and only 15% of slices were incorrectly predicted as non-ICH. The improvement in prediction accuracy demonstrates the effectiveness of the self-knowledge distillation method for inaccurate annotations. The above-mentioned results indicate that the main challenge is to distinguish blend sign from normal ICH. Our proposed auxiliary task (detection task) and self-knowledge distillation can partially solve this challenge.

### Visualizing what our model learns

**Figure 7:**
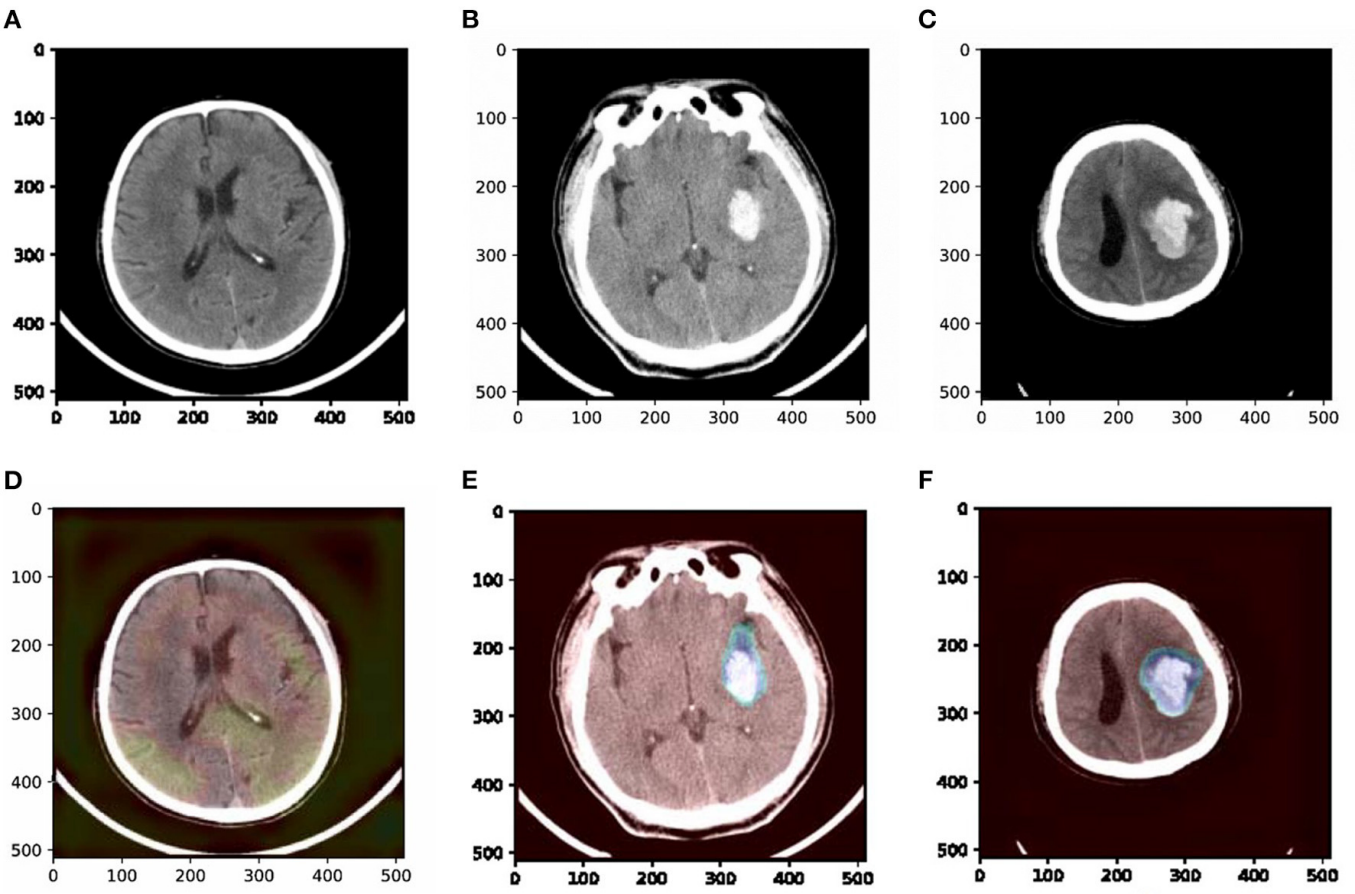
Examples of original slices with their corresponding class activation heatmaps. **(A)** Original slice of non-ICH, **(B)** original slice of normal ICH, **(C)** original slice of blend sign, **(D)** class activation heatmaps of the original slice of non-ICH, **(E)** class activation heatmaps of the original slice of normal ICH, and **(F)** class activation heatmaps of the original slice of blend sign. It is interesting to note that the regions with hemorrhage are strongly activated, and this is probably how the network can make decisions.

To understand which parts of a given image lead to the final classification decision, we apply “Class Activation Map” (CAM) visualization as a powerful technique (Ye et al., 2019). The specific method we choose is Grad-CAM (Selvaraju et al., 2017). CAM visualization produces heatmaps of “class activation” over input images. These heatmaps are 2D score grids, which indicate how important each location is regarding the class considered. CAM visualization is helpful to understand the decision process of our model as well as analyze the classification errors. It also might provide guidance for interpretation during clinical applications. Three examples from our dataset are shown in Figure 7, where the bright areas indicated high importance for decision marking and gray areas indicated low importance. It was interesting to note that the areas with bleeding attracted the most attention, while the areas without hemorrhage attracted less attention. In addition, we can also observe some non-overlapping regions between the highlighted regions and the bleeding regions. These results demonstrate that our approach could partly guide the model to pay more attention to the bleeding regions and misclassify some bleeding regions. The constraints of the detection task may cause misclassification. The detection task could only encourage the model to pay more attention to some rectangular areas rather than the accurate pixels.

## Discussion

This study focuses on the precise diagnosis of blend sign from head CT scans with a deep learning approach. Our contributions are summarized in the following aspects. First, we retrospectively collected 1,749 anonymous non-contrast head CT scans and annotated 13,276 slices for evaluation. Second, we present a prior knowledge-based model for blend sign from head CT scans. We apply an object detection task as the auxiliary task to help the model pay more attention to the regions with hemorrhage. Third, we propose a self-knowledge distillation strategy to reduce the impact of incorrect annotations. Results from Figure 6 and Table 3 confirm that the proposed auxiliary task (object detection task) and self-knowledge distillation indeed improved the performance of blend sign detection. Coarse heatmaps in Figure 7 show that the regions with hemorrhage attract more attention than the other regions. These heatmaps have the potential to be employed as a coarse bleeding localization map. In summary, our proposed algorithm assists the detection of blend sign and normal ICH with high accuracy, which may be a useful tool for the precise diagnosis of blend sign.

The proposed algorithm produces a pretty good performance in our dataset. AUCs for all the findings were >0.97. The F1 score for all the findings except the blend sign is >0.92. The *Specificity* for all the findings is greater than or equal to 0.94. For the diagnosis of blend sign, our algorithm achieves *Sensitivity* as 0.845, *Specificity* as 0.941, F1 score as 0.781, and AUC as 0.972. This may be because of two reasons. First, we retrospectively collected a large amount of anonymous non-contrast head CT scans from the First Affiliated Hospital of China Medical University. We apply the majority vote of three radiologists as the ground truth, which is a better gold standard than one radiologist. The quality of annotation is relatively high. Second, we apply an auxiliary task (object detection) and the self-knowledge distillation strategy, which are suitable for our condition. The auxiliary task could help the model to pay more attention to the regions with hemorrhage and extract more discriminate features. At the same time, self-knowledge distillation could vastly reduce the impact of incorrect annotations.

Our study also has several limitations. First, all slices in our dataset were from the Asian population, which may limit the generalization of our algorithm. It is desirable to include information on populations from other continents in the future. Second, to enhance our algorithm's performance and ensure there are enough positive samples to train the model. The prevalence of ICH (including blend sign and normal ICH) in our dataset is much higher than in some popular datasets and real clinical diagnoses. For example, the reported incidence rate of ICH is 12% in the famous Quer25k dataset (Chilamkurthy et al., 2018), while in our dataset, the incidence rate of ICH is 32%. The performance of our algorithm may change in real clinical applications. Third, we only have 1,614 available cases in our dataset, including 8,864 slices of non-ICH, 3,965 slices of normal ICH, and 447 slices of blend sign. The number of blend sign is quite limited. Performance may be adversely affected by the lack of training examples. Although transfer learning, auxiliary task, and self-knowledge distillation could boost the performance of our algorithm, the performance may drop a lot in real clinical cases. The next step is expanding our dataset and collecting more available scans, especially with blend sign. Finally, Fleiss's κ coefficient for blend sign is just 0.79, which means there is some inconsistency in the annotation of many slices with blend sign. The low blend sign identification rate of junior radiologists may need more investigation and may affect the training and generalization of our algorithm. Although self-knowledge distillation could partially alleviate the impact of inaccurate labeling, we also need to improve the reliability of annotations in the future.

## Conclusion

In this study, we propose a prior knowledge-based precise diagnosis of blend sign from head CT scans. We constructed a dataset with 1,614 available cases, 8,864 slices with non-ICH, 3,965 slices with normal ICH, and 447 slices with blend sign. To better distinguish the slices with normal ICH and blend sign, we propose the object detection task as an auxiliary task in addition to the classification task. The auxiliary task can help the model pay more attention to the region with hemorrhage. In addition, we employ a self-knowledge distillation strategy to reduce the influence of inaccurate annotations. Our precise diagnosis may assist less-experienced head CT interpreters in reducing initial misinterpretations. It also may reduce radiologists' workload and improve efficiency in a natural clinical setting. Experimental results show that our method achieved AUCs of 0.972 for blend sign, 0.978 for normal ICH, and 0.999 for non-ICH, which is a pretty good performance. In the future, we plan to collect more available scans with high reliability of annotations and extend our algorithm to clinical practice.

## Data availability statement

The raw data supporting the conclusions of this article will be made available by the authors, without undue reservation.

## Ethics statement

Written informed consent was obtained from the individual(s) for the publication of any potentially identifiable images or data included in this article.

## Author contributions

CW contribute to conceptualization, methodology, software, and original draft writing. JY contributes to data collection and annotation. JZ contributes to conceptualization, supervision, resources, and funding acquisition. SH contributes to conceptualization and data annotation. YQ contributes to software and validation. BF contributes to conceptualization and review. XL contributes to investigation and review. All authors contributed to the article and approved the submitted version.
